# Sr_5_(V^IV^OF_5_)_3_F(H_2_O)_3_ refined from a non-merohedrally twinned crystal

**DOI:** 10.1107/S1600536809019126

**Published:** 2009-05-23

**Authors:** Armel Le Bail, Anne-Marie Mercier, Ina Dix

**Affiliations:** a Laboratoire des Oxydes et Fluorures, CNRS UMR 6010, Université du Maine, 72085 Le Mans, France; b Bruker AXS GmbH, Östliche Rheinbrückenstrasse 49, D-76197 Karlsruhe, Germany

## Abstract

The title compound, penta­strontium tris­[penta­fluorido­oxido­vanadate(IV)] fluoride trihydrate, was obtained under hydro­thermal conditions. Its crystal structure has been refined from intensity data of a non-merohedrally twinned crystal. Two domains in almost equal proportions are related by a −180° rotation along the reciprocal [101]* vector. The structure may be considered as a derivative of the fluorite structure type, adopted here by SrF_2_. In the title compound, fluorite-like large rods are recognized, built up from a group of 16 Sr atoms of which 6 are substituted by V atoms, leading to [Sr_10_V_6_]_∞_ units. These rods extend infinitely along the *b* axis and are inter­connected by the three water mol­ecules. Each of the water mol­ecules is shared by two different Sr atoms belonging to two different rods. The rods are also inter­connected by an ‘independent’ F atom in a distorted triangular [FSr_3_] coordination and by hydrogen-bonding inter­actions *via* donor water mol­ecules. The acceptors are either F atoms or the O atoms of the vanadyl ion, VO^2+^, that is part of the [VOF_5_] isolated octa­hedron.

## Related literature

For V^IV^ in [VOF_5_] coordination, see: Crosnier-Lopez *et al.* (1994 ▶). Sr_2_V_2_^III^F_10_·H_2_O which was also synthesized during this study is isostructural with Sr_2_Fe_2_F_10_·H_2_O (Le Meins *et al.*, 1997 ▶). For a description of similar ’independent’ F atoms in the crystal structure of Sr_5_Zr_3_F_22_, see: Le Bail (1996 ▶). For bond-valence analysis, see: Brown & Altermatt (1985 ▶); Brese & O’Keeffe (1991 ▶).

## Experimental

### 

#### Crystal data

Sr_5_(VOF_5_)_3_F(H_2_O)_3_
*M* *_r_* = 996.97
Monoclinic, 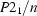

*a* = 11.217 (2) Å
*b* = 8.1775 (15) Å
*c* = 19.887 (4) Å
β = 105.999 (4)°
*V* = 1753.5 (6) Å^3^
*Z* = 4
Mo *K*α radiation
μ = 16.79 mm^−1^
*T* = 298 K
0.36 × 0.08 × 0.04 mm

#### Data collection

Bruker SMART APEX CCD area-detector diffractometer
Absorption correction: multi-scan (*TWINABS*; Bruker, 2003 ▶) *T*_min_ = 0.065, *T*_max_ = 0.553 (expected range = 0.060–0.511)
6596 measured reflections
6596 independent reflections
4299 reflections with *I* > 2σ(*I*)

#### Refinement

*R*[*F* ^2^ > 2σ(*F* ^2^)] = 0.038
*wR*(*F* ^2^) = 0.083
*S* = 0.93
6596 reflections
290 parameters
9 restraints
H atoms treated by a mixture of independent and constrained refinement
Δρ_max_ = 1.28 e Å^−3^
Δρ_min_ = −1.11 e Å^−3^

### 

Data collection: *SMART* (Bruker, 2003 ▶); cell refinement: *SAINT* (Bruker, 2003 ▶); data reduction: *SAINT*; program(s) used to solve structure: *SHELXS97* (Sheldrick, 2008 ▶); program(s) used to refine structure: *SHELXL97* (Sheldrick, 2008 ▶); molecular graphics: *DIAMOND* (Brandenburg, 1999 ▶) and *ORTEP-3* (Farrugia, 1997 ▶); software used to prepare material for publication: *publCIF* (Westrip, 2009 ▶).

## Supplementary Material

Crystal structure: contains datablocks I, global. DOI: 10.1107/S1600536809019126/wm2236sup1.cif

Structure factors: contains datablocks I. DOI: 10.1107/S1600536809019126/wm2236Isup2.hkl

Additional supplementary materials:  crystallographic information; 3D view; checkCIF report

## Figures and Tables

**Table 1:** Hydrogen-bond geometry (Å, °)

*D*—H⋯*A*	*D*—H	H⋯*A*	*D*⋯*A*	*D*—H⋯*A*
O1—H11⋯O4^i^	0.88 (4)	2.48 (5)	3.131 (6)	132 (5)
O1—H11⋯O5^i^	0.88 (4)	2.44 (4)	3.185 (6)	143 (5)
O1—H12⋯F4^ii^	0.882 (18)	1.96 (3)	2.796 (5)	157 (5)
O2—H21⋯F16	0.90 (4)	2.03 (4)	2.817 (5)	146 (5)
O2—H22⋯F7	0.92 (4)	2.18 (4)	2.905 (5)	135 (4)
O3—H31⋯F13	0.90 (5)	2.07 (5)	2.937 (5)	164 (5)
O3—H32⋯O6^iii^	0.89 (4)	2.17 (4)	2.873 (5)	135 (5)

Symmetry codes: (i) 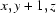; (ii) 
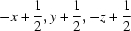
; (iii) 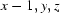.

**Table 2:** Valence-bond analysis of Sr_5_(V^IV^OF_5_)_3_F(H_2_O)_3_

	V1	V2	V3	Sr1	Sr2	Sr3	Sr4	Sr5	Σ	Σexpected
F1				0.38	0.34	0.35			1.07	1
F2			0.38	0.32			0.19	0.13	1.02	1
F3	0.51			0.26			0.19	0.18	1.14	1
F4		0.50			0.29			0.17	0.96	1
F5			0.46		0.25		0.13	0.25	1.09	1
F6			0.54			0.32	0.29		1.15	1
F7			0.52			0.29		0.16	0.97	1
F8	0.48			0.25			0.19	0.17	1.09	1
F9	0.51					0.09	0.16	0.19	0.95	1
F10	0.34			0.06			0.17;0.07	0.12;0.07	0.83	1
F11	0.55				0.08		0.17	0.12	0.92	1
F12		0.34		0.28			0.23	0.20	1.05	1
F13		0.46				0.22		0.20	0.88	1
F14		0.55			0.27		0.26		1.08	1
F15		0.65			0.32				0.97	1
F16			0.64	0.30					0.94	1
O1				0.22	0.21				2.03*	2
O2				0.14		0.27			2.01*	2
O3					0.17	0.24			2.01*	2
O4	1.66					0.17			1.83	2
O5		1.34				0.31			1.65	2
O6			1.29		0.25				1.54	2
Σ	4.05	3.84	3.83	2.21	2.18	2.26	2.05	1.94		
Σexpected	4	4	4	2	2	2	2	2		

Note: (*) adding a bond valence of 1.6 units, corresponding to the two H atoms linked to O1, O2 and O3, forming water molecules. The valence deficit observed on O4, O5 and O6, as well as on F4, F7, F13 and F16, is expected to be compensated by hydrogen bonding, since they behave as acceptors.

**Fig. 1.**
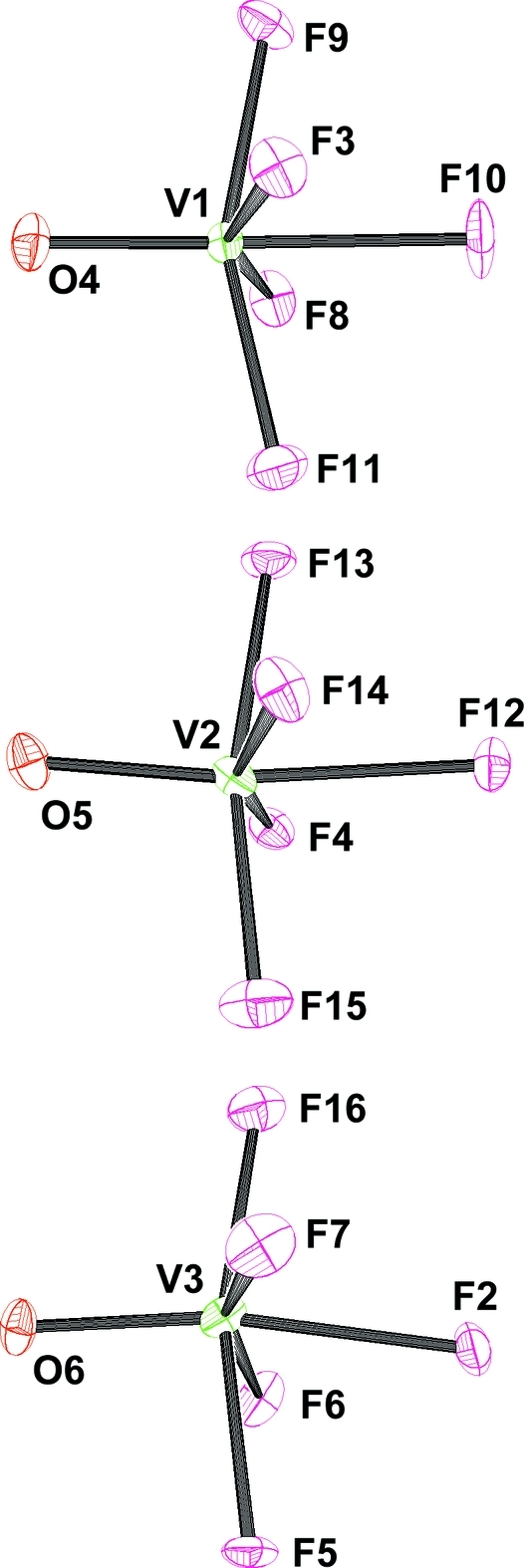
ORTEP-3 view of the [VIVOF5]3- octahedra showing the off-centered V position with short V=O distances (1.596–1.691 Å) and long opposite V—F distances (2.056–2.105 Å). Displacement ellipsoids are drawn at the 50% probability level.

**Fig. 2.**
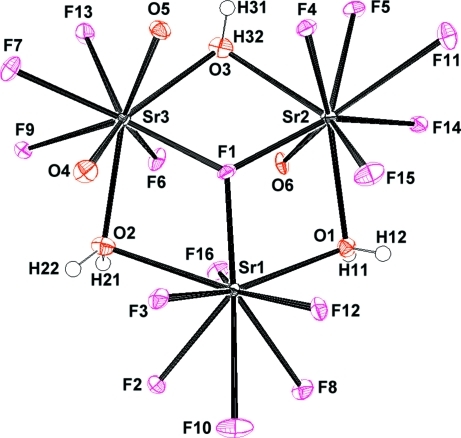
ORTEP-3 view of the three SrX9 (X = F,O,H2O) tri-capped trigonal prisms, sharing the F1 atom and two of the three water molecules. This shows how three different fluorite-like [Sr10V6] infinite rods are mainly interconnected. Displacement ellipsoids are drawn at the 50% probability level.

**Fig. 3.**
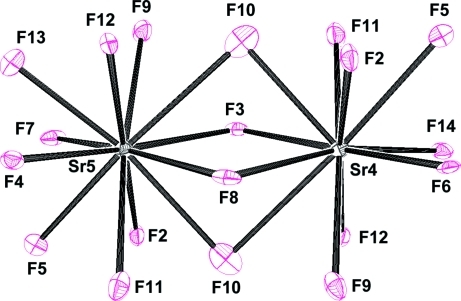
ORTEP-3 view of the Sr(5)F12 cuboctahedron and Sr(4)F11 truncated cuboctahedron associated by a face. This situation is like in the fluorite-type structure of SrF2. Displacement ellipsoids are drawn at the 50% probability level.

**Fig. 4.**
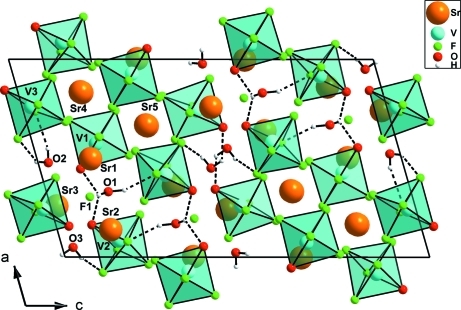
Crystal packing with view along [010] showing the isolated [VOF5] octahedra inside of fluorite-related [Sr10V6]∞ rods running along the b axis and delimited by the oxygen atoms of the vanadyl group, F1 atoms and the water molecules. Hydrogen bonds are indicated with dotted lines.

**Fig. 5.**
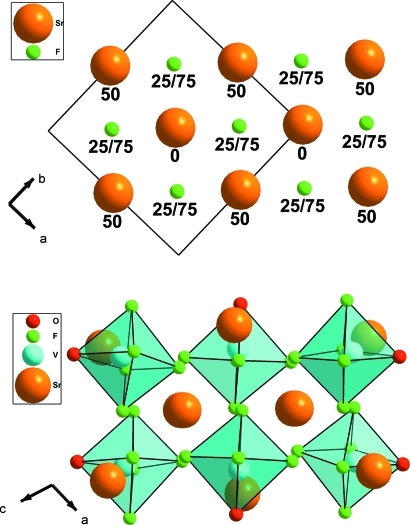
Comparison between the [Sr10V6]∞ rods in the title compound (bottom) and the corresponding Sr16 block in the SrF2 fluorite-type structure (top), obtained by doubling its c axis and reversely replacing V by Sr atoms. The heights of the z coordinates are indicated by the fractions×100.

**Fig. 6.**
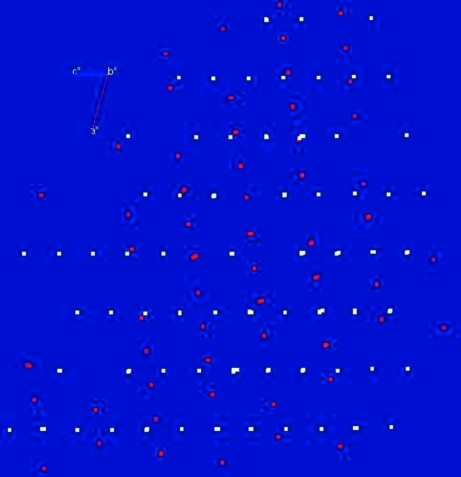
The two lattices of the two component crystal are visible. The SHELXL BASF parameter refines to 0.511 for this non-merohedral twin. The two domains are related by a rotation of -180° along the reciprocal [1 0 1]* vector.

## supplementary crystallographic information

### Comment

The title compound is the first hydrated strontium vanadium oxy-fluoride characterized crystallographically. It is built up from a network of SrO*_x_*F*_y_* polyhedra (*x* + *y* = 9 — 12), connected by faces, edges and vertices. Isolated (V^IV^OF_5_)^3-^ octahedra with a short V^IV^═O bond (1.596 (4)—1.691 (4) Å) characteristic of a vanadyl ion, VO^2+^, (Crosnier-Lopez *et al.*, 1994) are inserted into this network (Fig. 1). One of the fluorine atoms (F1) is shared exclusively by three strontium atoms (Sr1, Sr2, Sr3) in a triangular [FSr_3_] coordination, and will be named 'independent' according to previous descriptions (such a structure unit is also present in Sr_5_Zr_3_F_22_ (Le Bail, 1996); it shows the same *A*_5_*B*_3_*X*_22_ formula as the title compound). The three water molecules coordinate to these three (Sr1, Sr2, Sr3) strontium atoms, all of which have an overall ninefold coordination (Fig. 2), that is best described by a distorted tri-capped trigonal prism. The two remaining strontium atoms are exclusively coordinated by F atoms. The distorted [Sr(5)F_12_] cuboctahedron is connected to the [Sr(4)F_11_] polyhedra (best described as a defect cuboctahedron, lacking one vertex) by a square face (Fig. 3).

Any strong relation with the fluorite structure (adopted by SrF_2_) seems to be ruled out by the absence of F atoms in tetrahedral coordination [FSr_4_]. However, most F atoms are forming [FSr_3_V] distorted tetrahedra. One may consider that four strontium atoms (two Sr(4)F_11_ and two Sr(5)_12_ polyhedra sharing faces) represent a small fluorite structure relic around which half of the expected 12 Sr atoms are replaced by V atoms (forming [Sr_10_V_6_] blocks), which leads to the [FSr_3_V] distorted tetrahedra. Indeed, these blocks form infinite rods along the *b* axis, with formulation [Sr_5_V_3_]_∞_. The oxygen atoms of the six [VOF_5_] octahedra are part of the vanadyl V^IV^═O double bond and are all directed externally to these rods. This is well seen on the crystal structure projection (Fig. 4) where the water molecules are also placed in the rod interstices together with the F1 atom. Both types of ligands play a role in the interconnections between the rods. The relation of Sr_5_(V^IV^OF_5_)_3_F(H_2_O)_3_ with the SrF_2_ fluorite structure is provided in Fig. 5.

The hydrogen bonding involves both O and F atoms through Ow—H···O/F interactions (Table 1), participating in the interconnection of the [Sr_10_V_6_]_∞_ rods. One of these hydrogen bonds is clearly bifurcated (O1—H11···O4/O5). The distinction between F and O atoms was evident from the valence bond analysis (Table 2) according to the empirical expression given by Brown & Altermatt (1985), using parameters from Brese & O'Keeffe (1991). The valence bond analysis allows also to recognize the water molecules. The short vanadium-oxygen bond is characteristic of a vanadyl V^IV^═O double bond.

Thermal analysis (TGA) measurement from selected crystals shows a mass loss starting close to 573 K, without any clear stop for the expected 3H_2_O release; the corresponding 5.42% mass loss is attained at 693 K, then the mass loss accelerates and attains 17% up to 873 K. The X-ray powder diffraction pattern of the final product is similar to fluorite-type SrF_2_, but the real composition is more probably corresponding to a fluorite solid solution with formula close to Sr_5_V_3_^III^O_3_F_13_, *i.e.* (Sr/V)(O/F)_2_ which may be topotactically rebuilt from the title compound arrangement by re-aligning the fluorite-like [Sr_10_V_6_]_∞_ rods.

### Experimental

Hydrothermal growth at 493 K from (SrF_2_/VF_3_) in HF 5*M* or 1*M* solutions produced mixtures of pale-blue-green needle-like crystals (dominant in 5*M* solution), accompanied with polycrystalline Sr_2_V_2_^III^F_10_^.^H_2_O (dominant in 1*M* solution). The latter compound is orthorhombic, with cell parameters *a* = 7.8653 (4) Å, *b* = 19.9298 (7) Å, *c* = 10.7322 (6) Å (from X-ray powder data), space group *Cmca*, and is isostructural with Sr_2_Fe_2_F_10_^.^H_2_O (Le Meins *et al.*, 1997). All crystals of the title compound were found to be systematically affected by non-merohedral twinning (see Refinement section).

### Refinement

From a first data collection on a conventional four-circle diffractometer, the structure could be solved in spite of the twinning, removing a lot of reflections that belong to two domains, or that were partly overlapping. However, the final data/parameter ratio was so poor that a second data collection was performed (years later), using a Bruker *SMART APEX* system.

24642 single reflections were attributed to domain 1 (4436 unique), 24601 to domain 2 (4416 unique), and there were 11156 overlapping reflections attributed to both domains.

The reflections were integrated and processed into a HKLF5 file used for the refinement. The final refinement was based on the data set of domain 1. The *SHELXL* BASF parameter refined to 0.511. A view of the reflection spots of domains 1 and 2 is shown in Fig. 6.

The H atoms were located in Fourier difference maps and their positions could be refined freely. Because of the large spread of O—H and H—H distances, they were finally refined applying soft constraints (0.90 (2) Å for O—H). Their thermal parameters were fixed at 1.2 times that of the corresponding water oxygen atom.

The distinction between F and O atoms was clear from the valence bond analysis, allowing also to recognize the water molecules. The short vanadium-oxygen bond is characteristic of a vanadyl V^IV^=O double bond.

In the final Fourier map the highest peak is 0.85 Å from atom Sr1 and the deepest hole is 0.82 Å from atom Sr2.

### Crystal data

**Table:**

Sr_5_(VOF_5_)_3_F(H_2_O)_3_	*F*(000) = 1828
*M**_r_* = 996.97	*D*_x_ = 3.776 Mg m^−^^3^
Monoclinic, *P*2_1_/*n*	Mo *K*α radiation, λ = 0.71073 Å
Hall symbol: -P 2yn	Cell parameters from 323 reflections
*a* = 11.217 (2) Å	θ = 2.0–30.0°
*b* = 8.1775 (15) Å	µ = 16.79 mm^−^^1^
*c* = 19.887 (4) Å	*T* = 298 K
β = 105.999 (4)°	Needle, pale blue-green
*V* = 1753.5 (6) Å^3^	0.36 × 0.08 × 0.04 mm
*Z* = 4	

### Data collection

**Table:**

Bruker SMART APEX CCD area-detector diffractometer	6596 independent reflections
Radiation source: normal-focus sealed tube	4299 reflections with *I* > 2σ(*I*)
graphite	*R*_int_ = 0.0000
Detector resolution: 8.366 pixels mm^-1^	θ_max_ = 30.0°, θ_min_ = 1.9°
ω scans	*h* = −15→15
Absorption correction: multi-scan (TWINABS; Bruker, 2003)	*k* = 0→11
*T*_min_ = 0.065, *T*_max_ = 0.553	*l* = 0→27
6596 measured reflections	

### Refinement

**Table:**

Refinement on *F*^2^	Primary atom site location: structure-invariant direct methods
Least-squares matrix: full	Secondary atom site location: difference Fourier map
*R*[*F*^2^ > 2σ(*F*^2^)] = 0.038	Hydrogen site location: difference Fourier map
*wR*(*F*^2^) = 0.083	H atoms treated by a mixture of independent and constrained refinement
*S* = 0.93	*w* = 1/[σ^2^(*F*_o_^2^) + (0.0355*P*)^2^] where *P* = (*F*_o_^2^ + 2*F*_c_^2^)/3
6596 reflections	(Δ/σ)_max_ = 0.008
290 parameters	Δρ_max_ = 1.28 e Å^−^^3^
9 restraints	Δρ_min_ = −1.11 e Å^−^^3^
0 constraints	

### Special details

**Table:**

Geometry. All e.s.d.'s (except the e.s.d. in the dihedral angle between two l.s. planes) are estimated using the full covariance matrix. The cell e.s.d.'s are taken into account individually in the estimation of e.s.d.'s in distances, angles and torsion angles; correlations between e.s.d.'s in cell parameters are only used when they are defined by crystal symmetry. An approximate (isotropic) treatment of cell e.s.d.'s is used for estimating e.s.d.'s involving l.s. planes.
Refinement. Refinement of *F*^2^ against ALL reflections. The weighted *R*-factor *wR* and goodness of fit *S* are based on *F*^2^, conventional *R*-factors *R* are based on *F*, with *F* set to zero for negative *F*^2^. The threshold expression of *F*^2^ > σ(*F*^2^) is used only for calculating *R*-factors(gt) *etc*. and is not relevant to the choice of reflections for refinement. *R*-factors based on *F*^2^ are statistically about twice as large as those based on *F*, and *R*- factors based on ALL data will be even larger.

### Fractional atomic coordinates and isotropic or equivalent isotropic displacement parameters (Å^2^)

**Table:**

	*x*	*y*	*z*	*U*_iso_*/*U*_eq_	
Sr1	0.49725 (5)	0.91553 (6)	0.14704 (3)	0.01037 (10)	
Sr2	0.13869 (5)	0.91614 (7)	0.14686 (3)	0.01133 (11)	
Sr3	0.25819 (5)	0.58877 (7)	0.01760 (3)	0.01119 (11)	
Sr4	0.83170 (5)	1.15579 (6)	0.17512 (3)	0.01218 (12)	
Sr5	0.66417 (5)	1.16611 (6)	0.32847 (3)	0.01202 (12)	
V1	0.57273 (8)	0.41800 (11)	0.17307 (5)	0.00985 (18)	
V2	0.08398 (9)	0.41537 (12)	0.15922 (5)	0.01143 (19)	
V3	0.75847 (9)	0.92212 (12)	0.02341 (5)	0.0121 (2)	
F1	0.3050 (3)	0.7747 (4)	0.11547 (16)	0.0152 (7)	
F2	0.6850 (3)	0.9235 (4)	0.10740 (17)	0.0184 (7)	
F3	0.6078 (3)	0.6508 (3)	0.18699 (18)	0.0162 (7)	
F4	0.0792 (3)	0.6462 (4)	0.18369 (17)	0.0151 (7)	
F5	0.9049 (3)	0.8968 (4)	0.10573 (16)	0.0148 (7)	
F6	0.7705 (3)	1.1521 (4)	0.04540 (16)	0.0171 (8)	
F7	0.7368 (3)	0.6882 (4)	0.03245 (17)	0.0199 (8)	
F8	0.6052 (3)	0.1825 (3)	0.18916 (18)	0.0176 (7)	
F9	0.6924 (3)	0.4171 (4)	0.11824 (18)	0.0201 (7)	
F10	0.7406 (3)	0.4169 (4)	0.25298 (19)	0.0279 (8)	
F11	0.5154 (3)	0.4220 (4)	0.25564 (17)	0.0219 (8)	
F12	−0.0364 (3)	0.4062 (4)	0.22370 (17)	0.0157 (7)	
F13	−0.0774 (3)	0.4719 (4)	0.09201 (17)	0.0186 (8)	
F14	0.0383 (3)	0.1884 (4)	0.14929 (17)	0.0169 (8)	
F15	0.2141 (3)	0.3756 (4)	0.23846 (18)	0.0249 (9)	
F16	0.5983 (3)	0.9396 (4)	−0.03641 (17)	0.0211 (8)	
O1	0.3311 (4)	1.1241 (5)	0.1708 (2)	0.0156 (9)	
H11	0.314 (5)	1.207 (5)	0.1416 (19)	0.019*	
H12	0.339 (6)	1.144 (6)	0.2154 (11)	0.019*	
O2	0.4763 (4)	0.7121 (5)	0.0279 (2)	0.0202 (9)	
H21	0.482 (5)	0.790 (5)	−0.003 (2)	0.024*	
H22	0.547 (3)	0.650 (6)	0.038 (3)	0.024*	
O3	0.0503 (4)	0.7198 (5)	0.0304 (2)	0.0201 (10)	
H31	−0.002 (4)	0.653 (6)	0.044 (3)	0.024*	
H32	0.012 (4)	0.782 (6)	−0.006 (2)	0.024*	
O4	0.4460 (4)	0.4223 (5)	0.1120 (2)	0.0182 (9)	
O5	0.1632 (4)	0.4248 (5)	0.0991 (2)	0.0163 (9)	
O6	0.8434 (4)	0.9263 (5)	−0.03483 (19)	0.0145 (9)	

### Atomic displacement parameters (Å^2^)

**Table:**

	*U*^11^	*U*^22^	*U*^33^	*U*^12^	*U*^13^	*U*^23^
Sr1	0.0106 (3)	0.0085 (2)	0.0121 (3)	−0.0001 (2)	0.00308 (19)	−0.0003 (2)
Sr2	0.0092 (3)	0.0090 (2)	0.0150 (3)	0.0001 (2)	0.0020 (2)	0.0004 (2)
Sr3	0.0125 (3)	0.0076 (2)	0.0129 (3)	−0.0005 (2)	0.0026 (2)	−0.0011 (2)
Sr4	0.0118 (3)	0.0119 (3)	0.0125 (3)	−0.0007 (2)	0.0029 (2)	0.0001 (2)
Sr5	0.0119 (3)	0.0112 (3)	0.0129 (3)	−0.0004 (2)	0.0032 (2)	−0.0001 (2)
V1	0.0104 (5)	0.0067 (4)	0.0117 (5)	0.0003 (4)	0.0019 (4)	0.0005 (4)
V2	0.0095 (5)	0.0078 (4)	0.0166 (5)	0.0005 (4)	0.0030 (4)	−0.0012 (4)
V3	0.0165 (5)	0.0084 (4)	0.0101 (5)	0.0001 (4)	0.0017 (4)	0.0012 (4)
F1	0.0122 (17)	0.0094 (17)	0.0229 (18)	−0.0031 (13)	0.0030 (14)	−0.0049 (14)
F2	0.0170 (19)	0.0228 (18)	0.0194 (18)	−0.0064 (16)	0.0119 (15)	−0.0065 (16)
F3	0.0176 (19)	0.0070 (14)	0.023 (2)	−0.0006 (13)	0.0046 (15)	−0.0006 (14)
F4	0.0139 (19)	0.0122 (16)	0.0189 (19)	−0.0032 (14)	0.0037 (15)	−0.0027 (14)
F5	0.0097 (17)	0.0175 (17)	0.0132 (16)	−0.0003 (14)	−0.0037 (13)	0.0020 (14)
F6	0.031 (2)	0.0050 (15)	0.0157 (18)	0.0005 (15)	0.0078 (16)	0.0026 (13)
F7	0.028 (2)	0.0085 (16)	0.0213 (19)	0.0012 (14)	0.0038 (16)	0.0002 (14)
F8	0.018 (2)	0.0066 (15)	0.026 (2)	0.0014 (13)	0.0024 (15)	−0.0005 (14)
F9	0.0153 (18)	0.0225 (18)	0.0255 (19)	−0.0043 (16)	0.0105 (15)	−0.0083 (17)
F10	0.0141 (19)	0.037 (2)	0.024 (2)	0.0041 (18)	−0.0099 (15)	−0.0102 (18)
F11	0.026 (2)	0.0264 (19)	0.0156 (18)	−0.0051 (17)	0.0094 (15)	−0.0014 (16)
F12	0.0193 (19)	0.0149 (16)	0.0160 (17)	−0.0017 (15)	0.0100 (14)	0.0001 (15)
F13	0.0116 (19)	0.0253 (19)	0.0156 (18)	0.0044 (15)	−0.0017 (15)	−0.0046 (15)
F14	0.017 (2)	0.0093 (16)	0.027 (2)	−0.0020 (14)	0.0101 (16)	−0.0010 (14)
F15	0.025 (2)	0.025 (2)	0.020 (2)	0.0053 (16)	−0.0027 (17)	−0.0023 (15)
F16	0.021 (2)	0.021 (2)	0.0180 (18)	−0.0017 (15)	0.0009 (15)	0.0003 (15)
O1	0.017 (2)	0.016 (2)	0.013 (2)	−0.0004 (17)	0.0042 (18)	0.0000 (16)
O2	0.014 (2)	0.022 (2)	0.023 (2)	−0.0010 (18)	0.0036 (19)	0.0018 (19)
O3	0.017 (2)	0.019 (3)	0.023 (2)	0.0015 (18)	0.0039 (19)	0.0065 (19)
O4	0.016 (2)	0.017 (2)	0.018 (2)	0.0031 (19)	−0.0015 (17)	0.0026 (18)
O5	0.016 (2)	0.013 (2)	0.022 (2)	−0.0003 (18)	0.0098 (18)	0.0031 (18)
O6	0.019 (2)	0.013 (2)	0.014 (2)	0.0074 (18)	0.0085 (17)	0.0023 (17)

### Geometric parameters (Å, °)

**Table:**

V1—O4	1.596 (4)	Sr2—O3	2.768 (4)
V1—F11	1.921 (3)	Sr2—F11^ii^	2.932 (3)
V1—F9	1.949 (3)	Sr3—F1	2.411 (3)
V1—F3	1.948 (3)	Sr3—F6^i^	2.438 (3)
V1—F8	1.970 (3)	Sr3—F7^vi^	2.481 (3)
V1—F10	2.103 (3)	Sr3—O5	2.552 (4)
V2—O5	1.676 (4)	Sr3—F13^vii^	2.583 (3)
V2—F15	1.858 (4)	Sr3—O2	2.602 (4)
V2—F14	1.921 (3)	Sr3—O3	2.642 (4)
V2—F4	1.954 (3)	Sr3—O4	2.763 (4)
V2—F13	1.986 (3)	Sr3—F9^vi^	2.904 (3)
V2—F12	2.105 (3)	Sr4—F6	2.480 (3)
V3—O6	1.691 (4)	Sr4—F14^viii^	2.522 (3)
V3—F16	1.867 (4)	Sr4—F12^viii^	2.554 (3)
V3—F6	1.927 (3)	Sr4—F2	2.629 (3)
V3—F7	1.943 (3)	Sr4—F3^iv^	2.637 (3)
V3—F5	1.985 (3)	Sr4—F8^iii^	2.640 (3)
V3—F2	2.056 (3)	Sr4—F10^iv^	2.676 (4)
Sr1—F1	2.372 (3)	Sr4—F11^iv^	2.681 (3)
Sr1—F2	2.444 (3)	Sr4—F9^iii^	2.704 (4)
Sr1—F16^i^	2.468 (3)	Sr4—F5	2.773 (3)
Sr1—F12^ii^	2.487 (3)	Sr4—F10^iii^	2.978 (4)
Sr1—F3	2.513 (3)	Sr5—F5^iv^	2.536 (3)
Sr1—F8^iii^	2.525 (3)	Sr5—F13^ii^	2.611 (3)
Sr1—O1	2.663 (4)	Sr5—F12^ii^	2.613 (3)
Sr1—O2	2.851 (4)	Sr5—F9^iv^	2.630 (3)
Sr1—F10^iv^	3.062 (3)	Sr5—F3^iv^	2.662 (3)
Sr2—F1	2.418 (3)	Sr5—F8^iii^	2.668 (4)
Sr2—F15^ii^	2.441 (3)	Sr5—F4^ii^	2.678 (3)
Sr2—F4	2.475 (3)	Sr5—F7^iv^	2.688 (3)
Sr2—F14^iii^	2.501 (3)	Sr5—F2^iv^	2.780 (4)
Sr2—F5^v^	2.527 (3)	Sr5—F10^iii^	2.811 (4)
Sr2—O6^i^	2.629 (4)	Sr5—F11^iii^	2.816 (4)
Sr2—O1	2.685 (4)	Sr5—F10^iv^	2.978 (4)
			
O4—V1—F11	102.29 (18)	F4—V2—F13	81.95 (14)
O4—V1—F9	100.39 (18)	O5—V2—F12	172.52 (17)
F11—V1—F9	157.28 (15)	F15—V2—F12	87.89 (15)
O4—V1—F3	100.91 (18)	F14—V2—F12	80.22 (13)
F11—V1—F3	87.93 (15)	F4—V2—F12	79.42 (13)
F9—V1—F3	86.56 (15)	F13—V2—F12	78.43 (13)
O4—V1—F8	103.38 (18)	O6—V3—F16	100.78 (18)
F11—V1—F8	88.19 (15)	O6—V3—F6	96.80 (17)
F9—V1—F8	87.82 (15)	F16—V3—F6	93.72 (15)
F3—V1—F8	155.68 (13)	O6—V3—F7	101.05 (17)
O4—V1—F10	178.88 (19)	F16—V3—F7	90.65 (15)
F11—V1—F10	78.18 (15)	F6—V3—F7	160.49 (14)
F9—V1—F10	79.12 (15)	O6—V3—F5	94.15 (16)
F3—V1—F10	78.06 (14)	F16—V3—F5	165.02 (15)
F8—V1—F10	77.64 (14)	F6—V3—F5	85.67 (14)
O5—V2—F15	99.56 (18)	F7—V3—F5	85.24 (14)
O5—V2—F14	98.63 (17)	O6—V3—F2	169.75 (17)
F15—V2—F14	92.46 (15)	F16—V3—F2	89.22 (15)
O5—V2—F4	100.93 (17)	F6—V3—F2	80.15 (14)
F15—V2—F4	91.55 (15)	F7—V3—F2	80.90 (14)
F14—V2—F4	159.08 (14)	F5—V3—F2	75.92 (14)
O5—V2—F13	94.19 (17)	H11—O1—H12	118 (3)
F15—V2—F13	165.70 (15)	H21—O2—H22	109 (3)
F14—V2—F13	89.31 (15)	H31—O3—H32	113 (3)

Symmetry codes: (i) −*x*+1, −*y*+2, −*z*; (ii) −*x*+1/2, *y*+1/2, −*z*+1/2; (iii) *x*, *y*+1, *z*; (iv) −*x*+3/2, *y*+1/2, −*z*+1/2; (v) *x*−1, *y*, *z*; (vi) −*x*+1, −*y*+1, −*z*; (vii) −*x*, −*y*+1, −*z*; (viii) *x*+1, *y*+1, *z*.

### Hydrogen-bond geometry (Å, °)

**Table:**

*D*—H···*A*	*D*—H	H···*A*	*D*···*A*	*D*—H···*A*
O1—H11···O4^iii^	0.88 (4)	2.48 (5)	3.131 (6)	132 (5)
O1—H11···O5^iii^	0.88 (4)	2.44 (4)	3.185 (6)	143 (5)
O1—H12···F4^ii^	0.88 (2)	1.96 (3)	2.796 (5)	157 (5)
O2—H21···F16	0.90 (4)	2.03 (4)	2.817 (5)	146 (5)
O2—H22···F7	0.92 (4)	2.18 (4)	2.905 (5)	135 (4)
O3—H31···F13	0.90 (5)	2.07 (5)	2.937 (5)	164 (5)
O3—H32···O6^v^	0.89 (4)	2.17 (4)	2.873 (5)	135 (5)

Symmetry codes: (iii) *x*, *y*+1, *z*; (ii) −*x*+1/2, *y*+1/2, −*z*+1/2; (v) *x*−1, *y*, *z*.

### Table 2 Valence-bond analysis of Sr_5_(V^IV^OF_5_)_3_F(H_2_O)_3_

**Table:**

	V1	V2	V3	Sr1	Sr2	Sr3	Sr4	Sr5	Σ	Σexpected
F1				0.38	0.34	0.35			1.07	1
F2			0.38	0.32			0.19	0.13	1.02	1
F3	0.51			0.26			0.19	0.18	1.14	1
F4		0.50			0.29			0.17	0.96	1
F5			0.46		0.25		0.13	0.25	1.09	1
F6			0.54			0.32	0.29		1.15	1
F7			0.52			0.29		0.16	0.97	1
F8	0.48			0.25			0.19	0.17	1.09	1
F9	0.51					0.09	0.16	0.19	0.95	1
F10	0.34			0.06			0.17;0.07	0.12;0.07	0.83	1
F11	0.55				0.08		0.17	0.12	0.92	1
F12		0.34		0.28			0.23	0.20	1.05	1
F13		0.46				0.22		0.20	0.88	1
F14		0.55			0.27		0.26		1.08	1
F15		0.65			0.32				0.97	1
F16			0.64	0.30					0.94	1
O1				0.22	0.21				2.03*	2
O2				0.14		0.27			2.01*	2
O3					0.17	0.24			2.01*	2
O4	1.66					0.17			1.83	2
O5		1.34				0.31			1.65	2
O6			1.29		0.25				1.54	2
Σ	4.05	3.84	3.83	2.21	2.18	2.26	2.05	1.94		
Σexpected	4	4	4	2	2	2	2	2		

Note: (*) adding a bond valence of 1.6 units, corresponding to the two H atoms linked to O1, O2 and O3, forming water molecules. The valence deficit observed on O4, O5 and O6, as well as on F4, F7, F13 and F16, is expected to be compensated by hydrogen bonding, since they behave as acceptors.
